# Lithobius (Ezembius) ternidentatus sp. n. (Lithobiomorpha, Lithobiidae), a new species from China

**DOI:** 10.3897/zookeys.829.30884

**Published:** 2019-03-11

**Authors:** Sujian Pei, Haipeng Liu, Yanmin Lu, Xiaojie Hou, Huiqin Ma

**Affiliations:** 1 Institute of Myriapodology, School of Life Sciences, Hengshui University, Hengshui, Hebei 053000, China Hengshui University Hengshui China

**Keywords:** Centipede, Chilopoda, China, Hebei Province, myriapods

## Abstract

Lithobius (Ezembius) ternidentatus**sp. n.** (Lithobiomorpha, Lithobiidae), recently discovered from Wuyuezhai Mountain, Lingshou County, Shijiazhuang City, Hebei Province, China, is described. Morphologically it resembles L. (E.) multispinipes Pei, Lu, Liu, Hou, Ma & Zapparoli, 2016, but can be easily distinguished from the latter by having a different sized Tömösváry’s organ, different numbers of ocelli, obvious differences in ventral plectrotaxy of legs 14, and tarsal articulation ill-defined on legs 1–13, well-defined on legs 14–15. The main morphological characters of the known Chinese species of the subgenus Ezembius Chamberlin, 1919 based on adult specimens is presented.

## Introduction

*Ezembius* was originally proposed as a subgenus of *Lithobius* Leach, 1814 in the family Lithobiidae by Chamberlin (1919); it accommodates a group of 60 species/subspecies mostly known from Asia, with little extension into north-western North America. Known species colonize a wide range of habitats, from the Arctic and Subarctic to tropical and sub-tropical forests, to steppe and overgrazed stony areas of central Asia, to Himalayan montane forests, from the sea shore up to 5500 m (Himalayas) (Zapparoli and Edgecombe 2011, Qiao et al. 2018). Although the subgenus was formally proposed as new and described in 1923 (Chamberlin 1923), according to Jeekel (2005) its name had been already validated in 1919 (Chamberlin 1919). *Ezembius* is characterized by antennae with ca 20 articles; ocelli 1+4–1+20; forcipular coxosternal teeth usually 2+2; porodonts generally setiform, sometimes stout. Tergites are generally without posterior triangular projections; tarsal articulation of legs 1–13 is distinct. Female gonopods are with uni-, bi or tridentate claws, and 2+2–3+3 (rarely 4+4) spurs (Zapparoli and Edgecombe 2011).

The myriapod fauna of China is still poorly known and very little attention has been paid to the study of Lithobiomorpha, with only 82 species/subspecies hitherto known from the country. Altogether, 21 species of *Ezembius* have been recorded from China, but none of them have been reported from Hebei Province (Pei et al. 2018, Qiao et al. 2018). Here a new species, recently found in the Hebei Province, China, is described and illustrated. Tables of the main morphological characters of Chinese *Ezembius* species are presented.

## Materials and methods

All specimens were hand-collected under leaf litter or stones. The material was examined with the aid of a Motic-C microscope (Xiamen, China). The colour description is based on specimens preserved in 75% ethanol, and the body length is measured from the anterior margin of the cephalic plate to the posterior margin of the postpedal tergite. Type specimens are preserved in 75% ethanol and deposited in the School of Life Sciences, Hengshui University, Hengshui, China (HUSLS). The terminology of the external anatomy follows Bonato et al. (2010).

The following abbreviations are used in the text and the tables: **a**, anterior; **C**, coxa; **DaC spine**, anterior dorsal spine of coxa; **F**, femur; m, median; **p**, posterior; **P**, prefemur; **S**, **SS**, sternite, sternites; **T**, **TT**, tergite, tergites; **Ti**, tibia; **To**, Tömösváry’s organ; **Tr**, trochanter.

## Taxonomy

### Lithobiomorpha Pocock, 1895

#### Lithobiidae Newport, 1844

##### *Lithobius* Leach, 1814

###### Lithobius (Ezembius) Chamberlin, 1919

####### Lithobius (Ezembius) ternidentatus sp. n.

Taxon classificationAnimaliaLithobiomorphaLithobiidae

http://zoobank.org/CA3A868A-5684-4563-A942-F079371A4B9F

**Figure 1.**
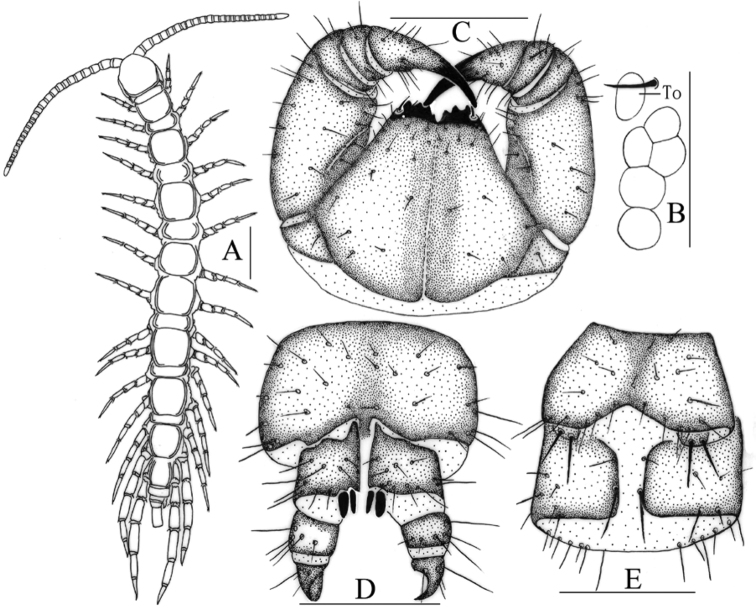
Lithobius (Ezembius) ternidentatus sp. n., holotype female and paratype male: **A** male habitus, dorsal view **B** male ocelli and Tömösváry’s organ (To), lateral view **C** female forcipular coxosternite, ventral view **D** female posterior segments and gonopods, ventral view **E** male: posterior segments and gonopods, ventral view. Scale bars: 2 mm (**A**); 200 μm (**B**); 250 μm (**C–E**).

Fig. 1A–E , Tables 1 , 2

######## Diagnosis.

Body length 7.1–8.5 mm, antennae commonly composed of 24 articles, but also 22+24 or 24+25, 5–6 ocelli on each side of head, arranged in two irregular rows, posterior two ocelli comparatively large; Tömösváry’s organ larger than the adjacent ocelli; commonly 3+3, but also 3+2 or 2+2 prosternal teeth, porodonts moderately slender, posterolateral to the lateral-most tooth, posterior angles of all tergites without triangular projections; coxal pore formula 3-4-4-3, oval to round, arranged in one row; female gonopods with 2+2 moderately small coniform spurs, apical claw simple; male gonopods short and small, with 1–3 long setae on the terminal segment.

######## Material examined.

**Holotype**: ♀ (Fig. 1), China, Hebei Province, Wuyuezhai Mountain, Lingshou County, Shijiazhuang City, 38°43'15.02"N, 114°08'32.62"E, 480 m, under litter of the forest floor in a mixed coniferous broad-leaved forest, 28 Sept 2014, leg. S. Pei, H. Ma. **Paratypes**: 33♀♀, 38♂♂, same data as holotype. **Other material**: 9♀♀, 6♂♂, China, Hebei Province, Shanyanggou, Longquanguan Town, Fuping County, Baoding City, 38°50'13.57"N, 114°03'26.93"E, 941 m, 7 Sept 2014, leg. S. Pei, H. Ma. Type specimens and other material are deposited in the HUSLS.

######## Description.

*Body* length: 7.1–8.5 mm, cephalic plate 0.75–0.97 mm long, 0.60–0.75 mm wide.

*Colour*: antennal articles and whole body pale yellow-brown, tergites darker, pleural region and sternites pale yellow with greyish hue; basal and proximal parts of forcipules, forcipular coxosternite, and SS XIV and XV darker.

*Antennae*: 22–25 articles, commonly 24 articles (Fig. 1A), 2 specimens 22+24, 3 specimens 24+25 articles; antennae articles length is approximately equal to width except basal articles II–V slightly longer than wide, distal-most article 2.7–3.1 times as long as wide; abundant setae on the antennal surface, less so on the basal articles, gradual increase in density of setae to about the fourth article, then more or less constant.

*Cephalic plate* smooth, convex, slightly wider than long; tiny setae emerging from pores scattered very sparsely over the whole surface; frontal marginal ridge with shallow anterior median furrow; short to long setae scattered along the marginal ridge of the cephalic plate; lateral marginal ridge discontinuous, posterior margin continuous, straight, wider than lateral marginal ridge (Fig. 1A).

Five or six oval to rounded ocelli on each side (Fig. 1B), most of them rounded, domed, translucent, usually darkly pigmented, situated in two irregular rows; the posterior two ocelli comparatively large; others subequal in size.

Tömösváry’s organ situated at anterolateral margin of the cephalic plate, about same size as the largest two ocelli and lying well apart from them (Fig. 1B).

Coxosternite subtrapezoidal (Fig. 1C), anterior margin narrow, lateral margins slightly longer than medial margins; median diastema moderately deep, narrow V-shaped; anterior margin with 3+3 acute triangular teeth, very few 2+2 (8% of studied individuals) or 2+3 (3% of studied individuals); porodonts slender, lying posterolateral to and separated from the lateral-most tooth (Fig. 1); scattered long setae on the ventral side of coxosternite, longer setae near the dental margin.

All tergites smooth, without wrinkles, dorsum slightly convex; tiny setae emerging from pores scattered sparsely over the entire surface, near the margin with few long setae; T I narrower posterolaterally than anterolaterally, generally trapezoidal, narrower than the cephalic plate and T III, obvious shorter than T III, the cephalic plate slightly wider than T III. Lateral marginal ridges of all tergites continuous. Posterior margin of TT I, III, V, and VII slightly concave, posterior marginal ridges continuous. Posterior margins of TT VIII, IX, XI, XIII, and XV concave, posterior marginal ridges discontinuous. Posterior angles of tergites generally rounded, without triangular projections. Miniscule setae scattered sparsely over the surface, 3–5 slightly thick and long setae on anterior and posterior angles of each tergite.

Posterior side of sternites narrower than anterior side, generally trapezoidal, smooth; setae emerging from sparsely scattered pores on the surface and lateral margin, few long setae on the surface of the anterior part of each sternite, 1–2 comparatively long setae scattered sparsely on the surface respective both of the middle part and posterior part of each sternite.

Legs robust, tarsal articulation ill-defined on legs 1–13, well-defined on legs 14–15; all legs with fairly long curved claws; legs 1–13 with anterior and posterior accessory spurs; anterior accessory spurs moderately long and slender, forming a moderately small angle with the claw, posterior accessory spurs slightly more robust, forming a comparatively large angle with the claw, legs 14 and 15 only with small posterior accessory spurs; long setae sparsely scattered over the surface of prefemur, femur and tibia of all legs, more setae on the tarsal surface; setae on dorsal surface of tarsus slightly shorter than the ventral, one row of thicker setae regularly arranged on the medial ventral side of tibia of legs 1–13, with setae significantly reduced in legs 14 and 15, no thicker setae regularly arranged in one row on the medial ventral side of tibia; legs 14 and 15 moderately thicker and longer than the anterior pairs in the female; leg plectrotaxy as in Table 1.

**Table 1.** Leg plectrotaxy of Lithobius (Ezembius) ternidentatus sp. n.

Legs	Ventral					Dorsal				
	C	Tr	P	F	Ti	C	Tr	P	F	Ti
1–9			mp	amp	am			ap	ap	ap
10			mp	amp	am	a		ap	ap	ap
11			mp	amp	am	a		amp	ap	ap
12			(a)mp	amp	am	a		amp	p	ap
13		m	amp	amp	am	a		amp	p	ap
14		m	amp	am	am	a		amp	p	p
15		m	amp	am	a	a		amp	p	

N.B. Letters in brackets indicate variable spines.

Coxal pores 3-3(4)-4(5)-3, commonly 3-4-4-3, round to slightly oval, in a row; coxal pore field set in a relatively shallow groove, the coxal pore-field fringe with prominence; prominence with short to moderately long setae sparsely scattered over the surface.

Female S 15 anterior margin broader than posterior, generally trapezoidal, posteromedially straight, colour yellow-brown; short to long sparse setae evenly scattered on surface; surface of the lateral sternal margin of genital segment well chitinized, posterior margin of genital sternite deeply concave between condyles of gonopods, except for a small, median tongue-shape bulge; relatively long setae sparsely scattered over ventral surface of the genital segment; gonopods: first article fairly broad, bearing 8–10 moderately long setae, arranged in three irregular rows; with 2+2 moderately long and slender, coniform spurs, inner spur slightly smaller than the outer; second article with 5–6 long setae, arranged in two irregular rows; third article with 3–4 comparatively long setae, arranged in one or two irregular rows; third article with a simple apical claw (Fig. 1D).

Male S 15 posterior margin narrower than anterior, posteromedially straight, sparsely covered with long setae on the surface; sternite of genital segment smaller than in female, usually well sclerotized, posterior margin deeply concave between the gonopods, without medial bulge; long setae sparsely scattered on the ventral surface of the genital segment, fewer setae near S 15, fringed with longer setae along the posterior margin; gonopods short, appearing as a small hemispherical bulge, with 1–3 long setae, apically slightly sclerotized (Fig. 1E).

######## Habitat.

The specimens here studied were collected in a mixed coniferous broad-leaved forest at ca 480–900 m above sea level, in moderately moist habitats under roadside stones and litter of the forest floor.

######## Etymology.

The specific name *ternidentatus* refers to the coxosternite anterior margin with 3+3 slightly acute triangular teeth.

######## Discussion.

The new species resembles L. (E.) multispinipes Pei, Lu, Liu Hou, Ma & Zapparoli, 2016 from the Xinjiang Autonomous Region in having 3+3 prosternal teeth commonly, the posterior two ocelli comparatively large, coxal pores 3–5 and with two coniform spurs on female gonopods. However, the new species can be easily distinguished by the following characters: the Tömösváry’s organ about same size as the largest ocellus in contrast to slightly smaller than the adjoining ocelli in L. (E.) multispinipes; and with five or six ocelli in new species instead of eight ocelli in L. (E.) multispinipes; and tarsal articulation ill-defined on legs 1–13 in the new species in contrast to well-defined on legs 1–13 in L. (E.) multispinipes; and legs 14 posterior accessory spur absent versus present in L. (E.) multispinipes, moreover, the 14 legs ventral plectrotaxy are obviously different: 0-1-3-2-2 compared to 0-1-3-2-1 in L. (E.) multispinipes.

To assist in the identification of the Chinese species of Lithobius (Ezembius), the main morphological characters (Table 2) of the known Chinese species of the subgenus Ezembius Chamberlin based on adult specimens are presented.

**Table 2.** The main morphological characters of the known Chinese species of subgenus Ezembius Chamberlin, 1919.

Characters	* anabilineatus *	* anasulcifemoralis *	* bidens *	* bilineatus *	* chekianus *	* datongensis *	* gantoensis *	* giganteus *	* insolitus *	* irregularis *
Authorities	Ma et al. 2015	Ma et al. 2013	Takakuwa 1939	Pei et al. 2014	Chamberlin and Wang 1952	Qiao et al. 2018	Takakuwa and Takashima 1949	Eason 1986	Eason 1993	Takakuwa and Takashima 1949
Distribution	China S (Guangxi)	China S (Guangxi)	China S (Taiwan)	China S (Guangxi)	China S (Zhengjiang and Taiwan)	China NW (Qinghai Province)	China NW (Shanxi)	China N (Inner Mongolia Autonomous region)	China S (Hong Kong)	China W (Shanxi)
Body length (mm)	11.9–12.1	10.1–12.3	15.0	9.0–9.1	16.0	12.3–14.2	9.0	15.0–50.0	10.0 –11.5	12.0
Number of antennal articles	23+23 articles in female, unkown in male	19+19–24+24, commonly 20+20	20–21	two specimens with 20+21, one specimen with 20+23	20+20	20+20	20–23	20+20	18+18 – 19+19	20+20
Number, arrangement and shape of the ocelli	5 – 6, in 2 rows	6, in 3 rows	7	5–6, in 2 rows	5, in 3 rows	10, in 3 rows	6	6–10, in 2–3 rows	6–8, in 2 rows	7, in 2 rows
Posterior ocellus	round, large	oval to round, large	comparatively large	oval to rounded	oval to round, comparatively large	comparatively large	oval to round, comparatively large	oval to round, comparatively large	oval to round, comparatively large	round, comparatively large
Seriate ocelli	subequal, all ocelli domed, translucent, usually darkly pigmented	one near ventral margin moderately small, others almost equal	not reported	subequal, all ocelli domed, translucent, usually darkly pigmented	not reported	not reported	comparatively large	not reported	not reported	subequal
Tömösváry’s organ	round, smaller than adjoining ocelli	moderately large, rounded, slightly larger than adjoining ocelli	at most same size as one ocellus	slightly larger than adjoining ocelli	not reported	slightly larger than nearest ocellus	subequal in size to adjoining medium large ocelli	slightly smaller than adjoining ocelli	slightly smaller than adjoining ocelli	same size as largest ocellus
Number and arrangement of coxosternal teeth	2+2, subtriangular	2+2, moderately blunt	2+2	2+2, slightly triangular	2+2	2+2 slightly acute	2+2, approximately sharp, small	2+2	2+2, approximately sharp, small	2+2, small
Porodont	long, lying posterolateral to lateral-most teeth	slender, lying posterolateral to lateral-most tooth, their base moderately bulged	moderately long	thick and long, lying posterolateral to lateral-most tooth	not reported	setiform porodonts separated from lateral tooth laterally	not reported	not reported	slender, lying posterolateral to lateral tooth, their base slightly bulged	long, their base slightly bulged
Tergites	smooth, backside slightly hunched	smooth	not reported	smooth, slightly hunched behind	not reported	almost smooth	smooth, without wrinkles	smooth, with slightly wrinkles	T1 smooth, other with wrinkles	smooth
Number of coxal pores	3–5, female 4454, 3554; male 4443, 4453	3–6, usually 4663, 5654, 5553, 5563 and 5565	5(6)555	usually females 4554, 5565; males 4553, 4454	6655 or 7665	4655 and 5575. Coxal pores 4654 and 4554 in male	3333	3333, 4554, 4555, 4565, 5565 or 5566	3–6, male 3443; female 4454, 4555, 5555, 5565	3–10, female 3–6 in 12^th^ leg, 4–6 in 13^th^ leg, 7–10 in 14^th^ and 15^th^ leg
Shape of coxal pores	round or slightly ovate	round or slightly ovate	round	ovate	not reported	rounded	round	round	round	round
Tarsus 1–tarsus 2 articulation on legs 1–13	not well-defined	not well-defined	Well-defined	not well-defined	not reported	distinct	not reported	Well-defined	not defined	Well-defined
Male 14^th^ leg	Obvious, thicker and stronger than other legs	markedly thicker and stronger than 1–13 legs, thicker and stronger than female	not reported	distinctly thick and strong	not reported	not reported	not reported	not reported	distinctly thick and strong	not reported
Male 15^th^ leg	obvious thicker and stronger than other legs	markedly thicker and stronger than 1–13 legs, thicker and stronger than female	not reported	distinctly thick and strong	not reported	not reported	not reported	not reported	distinctly thick and strong, with dark zones on dorsal of tibia	not reported
Dorsal sulci on male 14^th^ legs	absent	absent	not reported	with two, shallow longitudinal sulci	not reported	not reported	not reported	not reported	absent	not reported
Dorsal sulci on male 15^th^ legs	two distinct, shallow, dorsal sulci on femur and tibia	with a distinct, shallow, dorsal sulci on tibia	not reported	with two, shallow longitudinal sulci	not reported	not reported	not reported	not reported	absent	not reported
DaC spine	on 14^th^–15^th^ legs	on 14^th^–15^th^ legs	absent	on 4^th^–15^th^ legs	on 14^th^–15^th^ legs	on 12^th^–15^th^	absent	on 12^th^–15^th^ legs (on 11^th^ and 12^th^ legs sometimes present)	absent	on 13^th^–15^th^ legs
14^th^ accessory spur	anterior accessory spur reduced in size, only half length of posterior accessory spur	absent	not reported	anterior accessory spur absent	present	present	present	present	not reported	not reported
15^th^ accessory spur	absent	absent	not reported	anterior accessory absent	present	anterior accessory absent	present	absent	absent	not reported
Number and shape of spurs on female gonopods	2+2 moderately small, blunt, coniform spurs, inner spur slightly smaller than the outer	2+2 moderately blunt, with conical spurs, inner spur slightly smaller	3+3 or 4+4, sharp	2+2 moderately small, blunt, coniform spurs, inner spur slightly smaller than outer one	not reported	2+2 moderately large, coniform spurs	1+1, conical spurs	2+2	3+3, coniform spurs	2+2 or 2+3, moderately small, blunt, coniform spurs
Dorsal side of second article of female gonopods	with one spine lying dorsally on its external margin	no striking features	not reported	with three short, robust setae lying dorsally on its external margin	not reported	5-6 setae and five long curved spines	not reported	with eight spines in two irregular rows lying dorsally on its external margin	not reported	not reported
Apical claw of female gonopods (and lateral denticles)	simple, small subtriangular teeth in the inner	apical claw dimidiate	simple, small sharply teeth in the inner	apical claw bipartite, and its inner aspect broader	not reported	undivided, bearing a small triangular protuberance on ventral side	simple	simple	simple	simple and broad
Male gonopods	short and small bulge, with one to two long setae, apically slightly sclerotised	with a small bulge, without setae and apically less sclerotised	hemispherical, with two long setae	short and small bulge, having a long seta, apically slightly sclerotised	not reported	a hemispherical bulge, with three setae	not reported	not reported	not reported	not reported
Authoities	Pei et al. 2015	Qiao et al. 2018	Takakuwa 1939	Takakuwa 1940	Pei et al. 2016	Zapparoli 1991	Attems 1934	Attems 1927	Takakuwa and Takashima 1949	This paper	Pei et al. 2011	Pei et al. 2018
Distribution	China NW (Xinjiang Uygur)	China NW (Qinghai)	China S (Taiwan)	China (Taiwan, Sichuan, Jiangsu, Heilongjiang, Jilin, Liaoning)	China NW (Xinjiang Uygur)	China S (Taiwan)	China S (Fujian and Taiwan)	China S (Taiwan)	China W (Shanxi)	China N (Hebei)	China NW (Xinjiang Uygur)	China NW (Xinjiang Uygur)
Body length (mm)	9.6–13.3	17.0–18.0	18.0	22.0–23.0	11.6–22.6	16.0	15.0	Not reported	12.0	7.1–8.5	8.1－15.0	9.6–13.3
Number of antennal articles	19+19–21+21 commonly 20+20	20+20	19+19–21+21	20–28	commonly 20+20, (three specimens with 20+21, one specimen with 20+26 of 134 specimens)	20+20, 21+21	20+20 in female, 20+21 in male	19–22	20+20	22–25	20－24, commonly 20	19–22, commonly 20
Number, arrangement and shape of the ocelli	8–10, in 3 rows	11, in 3 rows	8–11, in 3 rows	9–13, in 3 rows	8, in 3 rows	3–4, in 1 or 2 rows	8, in 4 rows	7, in 2 rows	6	5–6, in 2 rows	10－13, in 3–4 rows	8–10, in 3 rows
Posterior ocellus	posterior two ocelli bigger than seriate ocelli	posterior ocellus largest	comparatively small	comparatively large	two ocelli large, oval to rounded	comparatively large	comparatively large	comparatively large	all ocelli same size	posterior two ocelli comparatively large	comparatively large	two ocelli comparatively large
Seriate ocelli	other seriate ocelli slightly larger than ocelli adjoining ventrally	not reported	not reported	same size	two near ventral margin moderately small, others almost equal	not reported	not reported	not reported	same size	others subequal in size	dorsal ones moderately large, those near ventral margin of ocellar field moderately small, others of moderate size	the adjoining Tömösváry organ slightly small
Tömösváry's organ	subequal in size to adjoining ocelli	smaller than adjacent ocelli	same size as adjoining ocelli	larger than adjoining ocelli	slightly smaller than adjoining ocelli	not reported	not reported	not reported	same size as ocelli	about same size as largest ocellus	slightly larger than adjoining ocelli	subequal in size to adjoining ocelli
Number and arrangement of coxosternal teeth	2+2, approximately blunt	3+2 blunt nipple-like teeth	2+2, comparatively large	2+2, small and sharp	3+3, slightly triangular	2+2	2+2	2+2	2+2, small and sharp	3+3 acute triangular, very few 2+2 or 2+3	2+2 moderately small and pointed	2+2 subtriangular slightly acute
Porodont	thick and long, lying posterolateral to lateral-most teeth	thick and strong separated from lateral tooth ventrolaterally	long and strong	lying posterolateral to lateral-most tooth	thick and long, lying posterolateral to lateral-most tooth	lying posterolateral to the lateral-most teeth	not obvious	not reported	slender and long	slender, lying posterolateral to, and separated from, lateral-most tooth	moderately thick in basal, moderately pointed, just posterolateral to lateral tooth	thick and strong, just posterolateral and separated from lateral tooth
Tergites	smooth, without wrinkles, backside slightly hunched	all smooth, without wrinkles	smooth	smooth, without wrinkles	smooth, without wrinkles and slightly hunched behind	smooth	with shallow wrinkles	Smooth, posterior angles slightly triangular in T14	not reported	smooth, without wrinkles, dorsum slightly convex	smooth, without wrinkles, backside slightly hunched	smooth, without wrinkles, dorsum slightly convex
Number of coxal pores	2–5, female commonly 4555, 4554, sometime 3454, 3455, 3343. male commonly 2332, 2333, sometime 3444, 3333	6555	6–7, usually 66(7)6	776(7)5(6)	3–5, 4555, 5555, 4444, 4455 (females) and 4444, 3344 (males)	3334	6554	4554	5555	3-3(4)-4(5)-3, commonly3-4-4-3	2–4, 3444, 3344, 3443, 3333 in female, and 3443, 2343, 2433, 2333 in male.	usually 4555, 4554, rarely 3454, 3455, 3343 in females and usually 2332, 2333, rarely 3444, 3333 in males
Shape of coxal pores	round or slightly ovate	circular	round to ovate	round or ovate	round to ovate	not reported	round	round	round	round to slightly oval	round or slightly ovate	round or slightly oval
Tarsus 1–tarsus 2 articulation on legs 1–13	not well-defined	well-defined	well-defined	well-defined	well-defined	not reported	not reported	well-defined	well-defined	ill-defined	well–defined	ill–defined
Male 14^th^ leg	remarkably thicker and stronger	moderately thicker and longer	not reported	not reported	thick and strong	not reported	not reported	not reported	thick and strong	moderately thicker and longer	moderately thicker and stronger	significantly thicker and stronger
Male 15^th^ leg	markedly thicker and stronger	moderately thicker and longer	not reported	not reported	thick and strong	not reported	femur and tibia thicker	femur and tibia thicker	thick and strong	moderately thicker and longer	thicker and stronger, with a circular protuberance on distal end of tibia	significantly thicker and stronger
Dorsal sulci on male 14^th^ legs	absent	absent	absent	not reported	absent	not reported	not reported	present on femur	present on femur and tibia	absent	absent	absent
Dorsal sulci on male 15^th^ legs	with a distinct, shallow, dorsal sulci on the tibia	absent	not reported	not reported	absent	not reported	not reported	present on femur and tibia	present on femur and tibia	absent	absent	present on femur
DaC spine	on 12^th^–15^th^ legs	on 13^th^–15^th^ legs, 12^th^ sometimes present	on 14^th^–15^th^ legs	on 12^th^–15^th^ legs	on 11^th^–15^th^ legs, 9^th^–10^th^ sometimes present	not reported	on 15^th^ legs present	on 15^th^ legs present	absent	on 10^th^–15^th^ legs	on 13^th^–15^th^ legs, 12^th^ sometimes present	on 12^th^–15^th^ legs
14^th^ accessory spur	present	present	present	not reported	present	not reported	not reported	not reported	not reported	anterior accessory spur absent	present	present
15^th^ accessory spur	anterior absent	absent	present	not reported	absent	not reported	absent	not reported	not reported	anterior accessory spur absent	absent	absent
Number and shape of spurs on female gonopods	3+4, or 4+4 small, blunt, coniform spurs, commonly with 3+3, inner spur smaller than outer one	2+2 moderately long, bullet-shaped spurs inner spur slightly smaller and more anterior than outer one	3+3 moderately sharp, slender conical spurs	3+3, same size	2+2, blunt, coniform spurs, with inner spur smaller than outer one	2+2	2+2, slender	2+2, thick spurs	2+2, strong, long and sharp	2+2 moderately long and slender, coniform	2ﬂ 2 moderately long, coniform spurs, inner spur slightly smaller and more anterior than outer	3+3, few 3+4, only one 4+4 coniform spurs
dorsal side of the second article of female gonopods	with three long setae lying dorsally on its anterior external margin	three long setae along dorsolateral ridge	not reported	not reported	with 3–4 long setae and 5–6 spines lying dorsally on its external margin	not reported	not reported	not reported	not reported	no setae and spines	three spurs arranged in one irregular row on dorsal terminal part	3 long setae and four short, robust spines lying dorsally on posterior part of external margin
Apical claw of female gonopods (and lateral denticles)	simple and broad	simple, having small triangular protuberance on ventral side	simple	simple	simple	simple	simple	dimidiate	simple	simple	broad, and tridentate	simple, with a very small subtriangular blunt denticle on inner margin
Male gonopods	small bulge, with one to two long setae apically slightly sclerotised	small, semicircular article with 3-5 seta on its surface	hemispherical bulge,	without setae	hemispherical bulge, having a long seta, and apically slightly sclerotised	not reported	not reported	not reported	not reported	short, small hemispherical bulge, with 1–3 long setae, apically slightly sclerotized	small bulge, with 1–2 long setae on surface, and terminal slightly sclerotised	small hemispherical bulge, with 1–2 long setae

## Supplementary Material

XML Treatment for Lithobius (Ezembius) ternidentatus
